# Alcohol Induced Hepatic Degeneration in a Hepatitis C Virus Core Protein Transgenic Mouse Model

**DOI:** 10.3390/ijms15034126

**Published:** 2014-03-07

**Authors:** Dong-Hyung Noh, Eun-Joo Lee, Ah-Young Kim, Eun-Mi Lee, Chang-Woo Min, Kyung-Ku Kang, Myeong-Mi Lee, Sang-Hyeob Kim, Soo-Eun Sung, Meeyul Hwang, Dae-Yeul Yu, Kyu-Shik Jeong

**Affiliations:** 1 College of Veterinary Medicine, Kyungpook National University, 80 Daehakro, Buk-gu, Daegu 702-701, Korea; E-Mails: ndh1122@hanmail.net (D.-H.N.); miffy525@hanmail.net (E.-J.L.); pretersensual@hanmail.net (A.-Y.K.); nikeun@hanmail.net (E.-M.L.); mangelos@hanmail.net (C.-W.M.); qzpmqzpm@naver.com (K.-K.K.); 2myeongmi@naver.com (M.-M.L.); laversang@naver.com (S.-H.K.); sse8679@naver.com (S.-E.S.); meeyul@hanmail.net (M.H.); 2 Stem Cell Therapeutic Research Institute, Kyungpook National University, 80 Daehakro, Buk-gu, Daegu 702-701, Korea; 3 Korea Research Institute of Bioscience and Biotechnology, Daejon 305-333, Korea; E-Mail: dyyu10@kribb.re.kr

**Keywords:** hepatitis C virus, alcohol, HCV core protein, TG-99

## Abstract

Hepatitis C virus (HCV) has become a major public health issue. It is prevalent in most countries. HCV infection frequently begins without clinical symptoms, before progressing to persistent viremia, chronic hepatitis, cirrhosis and hepatocellular carcinoma (HCC) in the majority of patients (70% to 80%). Alcohol is an independent cofactor that accelerates the development of HCC in chronic hepatitis C patients. The purpose of the current study was to evaluate ethanol-induced hepatic changes in HCV core-Tg mice and mutant core Tg mice. Wild type (NTG), core wild-Tg mice (TG-K), mutant core 116-Tg mice (TG-116) and mutant core 99-Tg mice (TG-99) were used in this investigation. All groups were given drinking water with 10% ethanol and 5% sucrose for 13 weeks. To observe liver morphological changes, we performed histopathological and immunohistochemical examinations. Histopathologically, NTG, TG-K and TG-116 mice showed moderate centrilobular necrosis, while severe centrilobular necrosis and hepatocyte dissociation were observed in TG-99 mice with increasing lymphocyte infiltration and piecemeal necrosis. In all groups, a small amount of collagen fiber was found, principally in portal areas. None of the mice were found to have myofibroblasts based on immunohistochemical staining specific for α-SMA. CYP2E1-positive cells were clearly detected in the centrilobular area in all groups. In the TG-99 mice, we also observed cells positive for CK8/18, TGF-β1 and phosphorylated (p)-Smad2/3 and p21 around the necrotic hepatocytes in the centrilobular area (*p* < 0.01). Based on our data, alcohol intake induced piecemeal necrosis and hepatocyte dissociation in the TG-99 mice. These phenomena involved activation of the TGF-β1/p-Smad2/3/p21 signaling pathway in hepatocytes. Data from this study will be useful for elucidating the association between alcohol intake and HCV infection.

## 1. Introduction

Hepatocellular carcinoma (HCC) is a cause of systemic disorders and fatal human disease [1]. Several agents are strongly associated with the development of HCC including chemicals such as aflatoxin B1 and alcohol as well as viruses such as hepatitis B virus (HBV) and hepatitis C virus (HCV) [2–4]. HCV is the major etiologic agent of chronic hepatitis, a disease that is closely linked to the development of HCC in many countries [2,5].

Most cases of HCV infection start without clinical symptom before progressing to persistent viremia, chronic hepatitis, hepatic cirrhosis and HCC [2]. Generation of HCV core transgenic mice has demonstrated the major role of core protein in HCC progression. These mice present chronic steatosis and develop HCC [6]. Additionally, HCV core protein has been shown to induce apoptosis in mice [6] and transformed cell line [7,8]. Numerous previous studies have suggested that chronic ethanol supply results in hepatic fatty change, fibrosis and hepatic cirrhosis in animal models through the induction of hypoxia, ethanol metabolite accumulation, DNA modification with acetaldehyde adduct formation, impairment of nutrient uptake and induction of cytochrome P4502E1 (CYP2E1) [9,10]. CYP2E1 and hemeprotein can generate reactive oxygen species (ROS) through the reduction of dioxygen to superoxide anions that leads to production of hydrogen peroxide and hydroxyl radical [5,11,12]. ROS are endogenous oxygen-containing molecules formed as normal products during aerobic metabolism [12]. ROS include a number of species such as superoxide, hydroxyl, peroxyl radicals and certain nonradicals such as singlet oxygen and hydrogen peroxide that can be easily converted into radicals. ROS can induce genetic mutations as well as chromosomal alterations, and thus promote carcinogenesis [13].

In cases of chronic liver disease, alcoholism and HCV infection frequently coexist [14]. It is widely believed that the combination of ethanol and chronic HCV infection increases viral replication, impairs cellular immunity and finally results in severe and progressive liver disease [15]. Previously, a synergistic effect was observed between the core protein and alcohol administration with respect to the generation of hydroperoxide in mouse liver [16]. This is of great interest because a synergistic effect between excessive alcohol intake and HCV infection has been documented for the development of HCC in chronic hepatitis C patients [17]. Alcohol is also known to be an independent cofactor that accelerates the development of HCC in patients with chronic hepatitis C [14].

Over the past decade, genetically engineered mouse models have been used to study the mechanisms of hepatitis C [18]. Recently three transgenic mouse lines were created expressing the HCV core gene and its mutants in liver [19]. One is the transgenic mice model, which has a wild type core (TG-K). Another is the transgenic mice model in which serine116 was replaced by isoleucine (TG-116) and the other is that in which serine 99 was substituted with glutamine (TG-99). Both Serine116 and Serine 99 is phosphorylation site of HCV core protein. The serine 99 is phosphorylated by PKC, while serine 116 is phosphorylated by PKA [20].

In this study, we determined whether alcohol administration may have a synergistic effect on pathological changes in the liver of these transgenic mice. Also, we elucidated the function of each phosphorylation site under the hepatic injury like alcohol administration by using these transgenic mice whose phosphorylation site is substituted by other amino acids.

## 2. Results

### 2.1. Histopathological Changes in the HCV Core Tg-Mice

**Figure 1.**
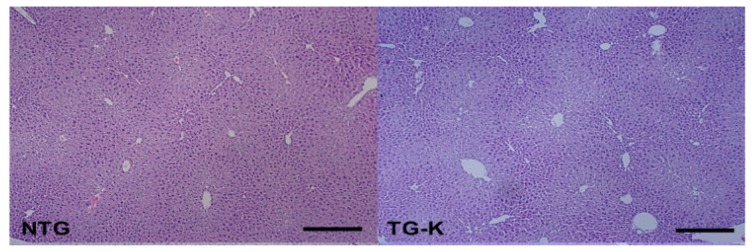

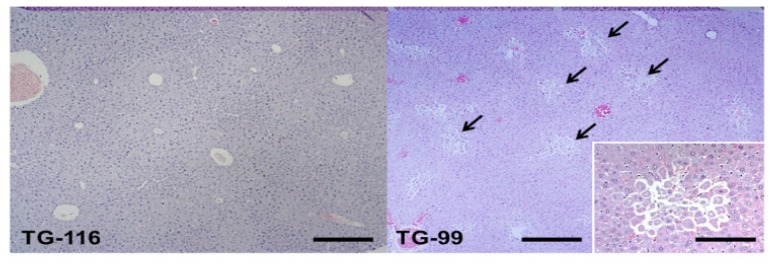
Representative microphotograph of livers stained with hematoxylin and eosin (H&E) of NTG, TG-K, TG-116 and TG-99. Moderate centrilobular necrosis was observed in the liver of NTG, TG-K and TG-116. Severe centrilobular necrosis was observed in the TG-99. Arrows indicate centrilobular necrosis. NTG is wild type mice; TG-K is transgenic mice which have wild type core; TG-116 is transgenic mice whose serine116 was replaced by isoleucine; TG-99 is transgenic mice in which serine 99 was substituted with glutamine. Scale bar = 200 μm.

Hepatic fatty change and necrosis were successfully induced with ethanol in all groups. Moderate centrilobular necrosis was detected in the liver of the non-transgenic mice (NTG) and TG-K (Figure 1). Hepatic cord destruction and moderate centrilobular necrosis were evident in the TG-116 in which many hepatocytes had a homogeneous cytoplasm and were mildly dissociated (Figure 1). Severe centrilobular necrosis and hepatocyte dissociation were observed in TG-99 with severe lymphocyte infiltration and piecemeal necrosis (Figure 1). Our results suggest that lymphocyte infiltration, piecemeal necrosis and hepatocyte dissociation may be associated with mutation in the residue 99 of core protein from serine to glutamine.

### 2.2. Evaluation of Collagen Production

**Figure 2.**
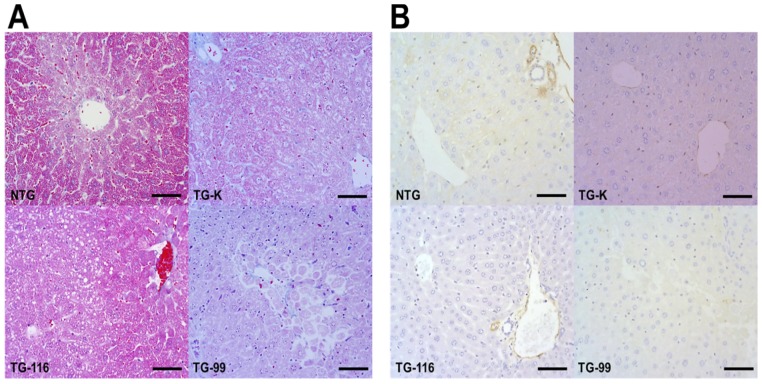
(**A**) Azan-Mallory staining. Scale bar = 50 μm; (**B**) Immunohistochemistry staining of α-Smooth muscle actin (α-SMA). Scale bar = 50 μm.

To detect collagen fibers in the liver, Azan-Mallory staining was performed. A small amount of collagen fiber that is stained blue with Azan-Mallory staining was detected in the portal areas (Figure 2).

**Figure 3.**
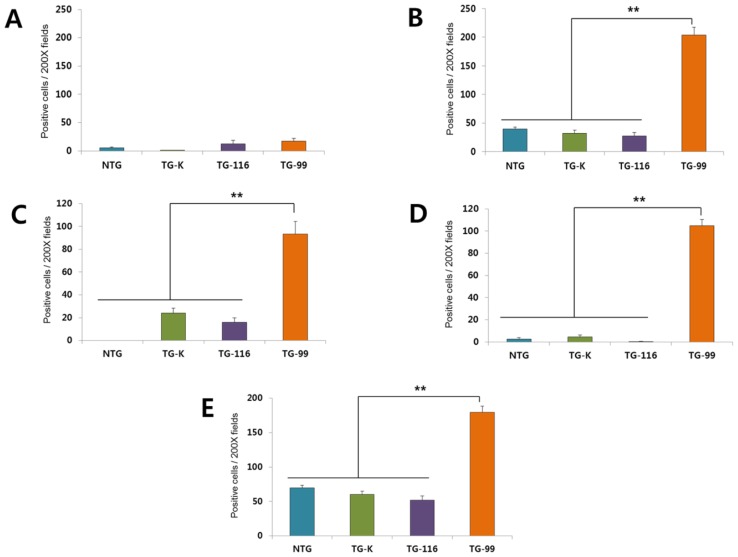
Quantification of the positive cells for each immunohistocytochemistry staining in the 200X magnification. (**A**) alpha-SMA; (**B**) CK8/18; (**C**) CYP2E1; (**D**) TGF-beta; (**E**) Psmad2/3. For this, positive cells for each staining were counted on five selected microscopic fields in each group. The data are showed as the means ± S.E.M. Statistical analysis was performed using one way ANOVA (** *p* < 0.01).

Expression of α-Smooth muscle actin (α-SMA) was also evaluated to identify myofibroblasts (MFBs) that are the main source of collagen production in case of liver cirrhosis. In this study, α-SMA was not detected, indicating no myofibroblast was present in the liver of the mice (Figures 2 and 3A).

### 2.3. Identification of Marker for Alcoholic Hepatitis in HCV Core Tg-Mice

**Figure 4.**
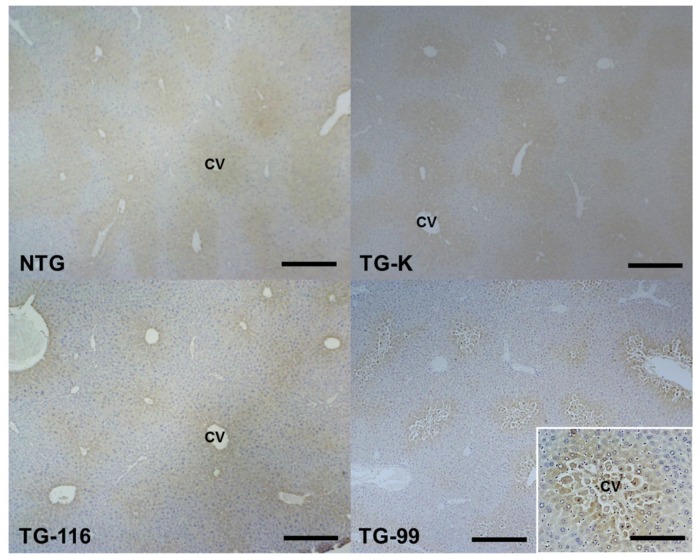
Immunohistochemistry staining of CYP2E1. All groups showed CYP2E1 positive cells in the centrilobular area. More CYP2E1 positive cells were observed in the TG-99. Scale bar = 200 μm. Scale bar in the inset = 50 μm. CV, central vein.

CYP2E1 gene is critical for ethanol metabolism in the liver [10,21]. CYP2E1 expression in the liver of all mice was evaluated via immunohistochemical staining. CYP2E1-positive cells were observed in the centrilobular area in all mice. Of particular interest, significantly more CYP2E1-positive cells were detected in the TG-99 compared to other groups (Figures 3C and 4).

**Figure 5.**
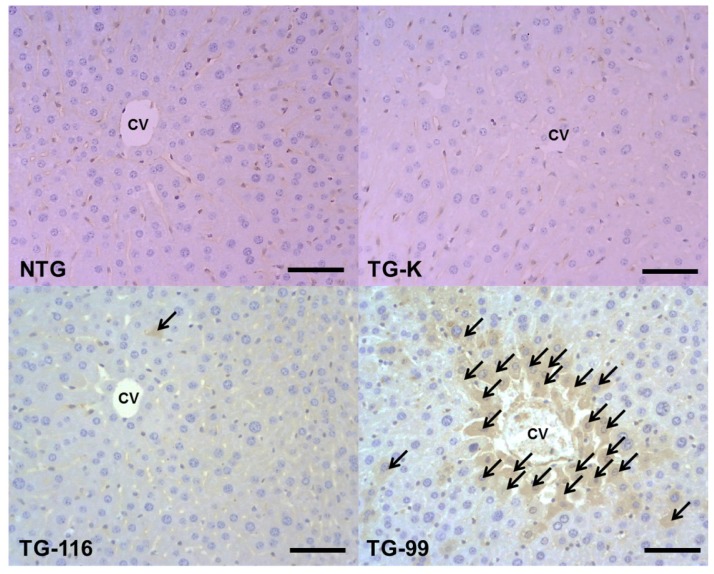
Immunohistochemistry staining of CK8/18. Arrows indicate CK8/18 positive cells. Scale bar = 200 μm. CV, central vein.

To detect Mallory body (MB) which is a marker of hepatitis and fibrosis [22], CK8/18 expression was quantified by immunohistochemistry. In the TG-99 mice, CK8/18-positive cells were markedly observed in the cytoplasm of dissociated and necrotic hepatocytes in the centrilobular area. In contrast, no CK8/18-positive cell was found in NTG, TG-K and TG-116 (Figures 3B and 5).

### 2.4. TGF-β1 Signaling Cascade in HCV Core Tg-Mice

**Figure 6.**
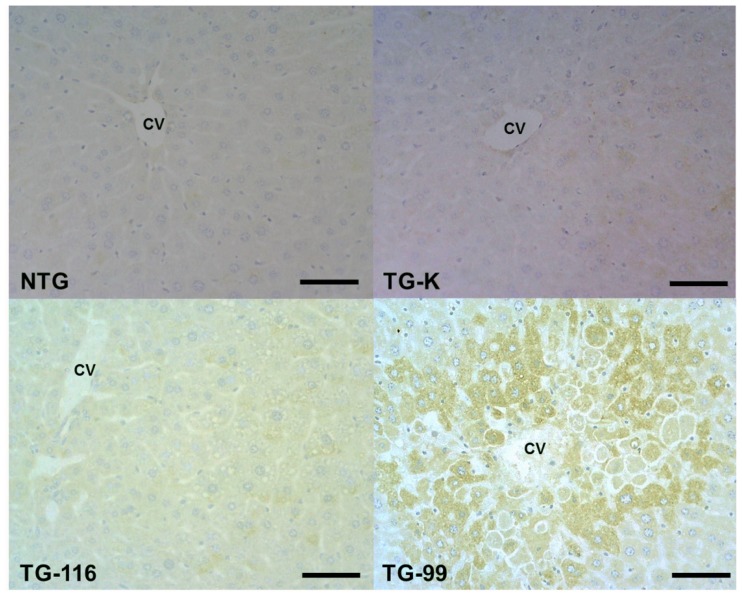
Immunohistochemistry staining of TGF-β1. Scale bar = 50 μm. CV, central vein.

TGF-β1 is a clinical marker of HCV infection and HCC progression since its level increases during HCV infection and progression of HCV-associated HCC [7]. TGF-β1 positive cells were predominantly detected cytoplasm of TG-99 around centrilobular necrosis. Otherwise, there were only a few TGF-β1-positive cells in NTG, TG-K and TG-116 (Figures 3D and 6).

**Figure 7.**
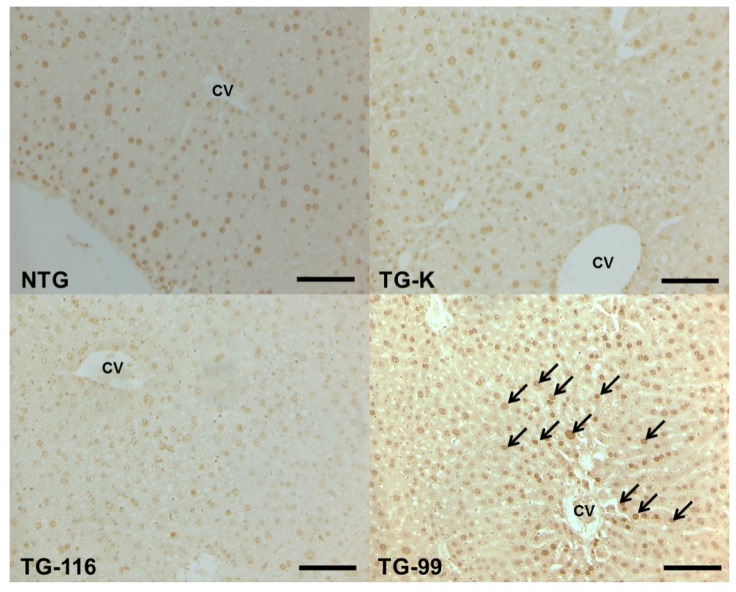
Immunohistochemistry staining of p-SMAD2/3. Arrows indicate p-SMAD2/3 positive cells. Scale bar = 100 μm. CV, central vein.

Smad2/3 is an immediate intracellular signaling molecule belonging to the TGF-β superfamily. This factor plays an important role in the activation of hepatic stellate cells and hepatic fibrosis [23,24]. p-Smad2/3-positive cells were most prominent in the TG-99 around dissociated and necrotic hepatocytes in the centrilobular area (Figures 3E and 7). Many p-Smad2/3-positive cells were observed in the TG-K, but not as many as TG-99. Mild level of p-Smad2/3-positive cells were also detected in the NTG and TG-116.

**Figure 8.**
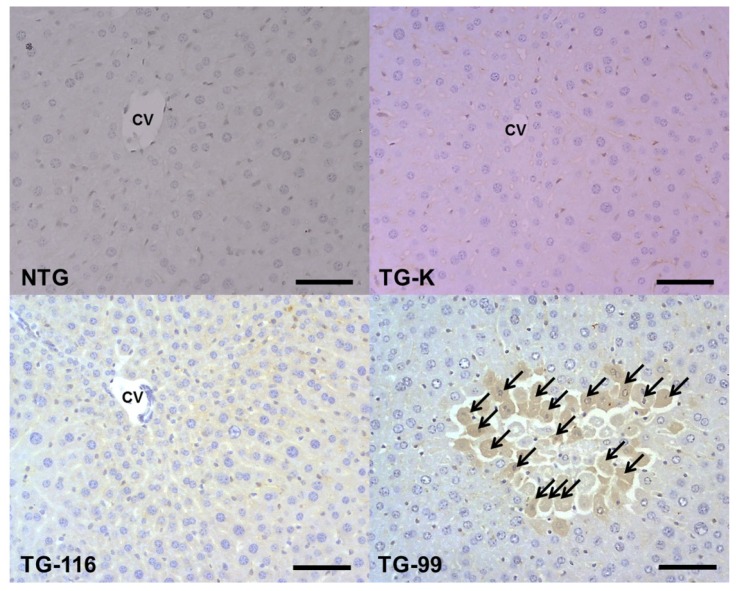
Immunohistochemistry staining of p21. In the TG99, there were lots of p21 positive hepatic cells near central veins. Arrows indicate p21 positive cells. Scale bar = 50 μm. CV, central vein.

p21 protein is a universal inhibitor of cyclin-dependent kinases (CDK) and is regulated transcriptionally by p53, which is activated by DNA stress. Smad transactivation of *p21^WAF1^* gene occurs in proximal promoter region. p21 antigen was detected primarily in the nuclei of hepatocytes and mononuclear cells. p21-positive cells were markedly detected around dissociated and necrotic hepatocytes in centrilobular area in TG-99 (Figure 8). However, there was no p21-positive cell in NTG, TG-K and TG-116 (Figure 8).

## 3. Discussion

Alcohol ingestion is known to cause a variety of pathological alterations in the livers of both humans and experimental animals. These changes are due to cellular metabolic disturbances associated with ethanol oxidation and oxidative stress [10,16]. Ethanol-induced oxidative stress plays a major role in mechanism underlying hepatic injury. Many pathways are thought to contribute to the ability of ethanol to induce oxidative stress [16]. One of the most promising pathways is induction of the CYP2E1 form of cytochrome P450 by ethanol. CYP2E1 is an effective generator of ROS such as superoxide anion radicals and hydrogen peroxide. In the presence of iron catalysts, CYP2E1 produces powerful oxidants such as hydroxyl radicals [25]. Oxidative stress is increased in both patients with alcoholic liver disease and individuals infected with HCV. Liver injury caused by oxidative stress might be due to the effects of viral proteins on host cellular functions. Previous studies showed that the HCV core protein and non-structural 5A (NS5A) protein interact with multiple intracellular structures including lipid droplets, endoplasmic reticulum, and mitochondria *in vitro* and *in vivo* [26,27]. HCV core protein increases the generation of ROS by interfering with mitochondrial electron transport. Other sources of ROS include macrophages and inflammatory cells as well as hepatocytes [13]. Furthermore, induction of the microsomal pathway involving CYP2E1 contributes to increased acetaldehyde generation, protein adduct formation, enzyme inactivation, decreased DNA repair, reduced liver glutathione (GSH) depletion, free radical-mediated toxicity, and lipid peroxidation [21]. In the current study, CYP2E1 expression was observed via immunohistochemical staining of liver sections from all the experimental groups, indicating that ethanol consumption was conducted successfully in all the experimental groups.

In most cases, hepatitis is preceded by hepatic cirrhosis. Many cirrhotic patients will have had a liver biopsy that shows the morphological features of alcoholic hepatitis, defined by an international group of hepatologists as characterized by hepatocyte necrosis and a neutrophil infiltration, usually with Mallory bodies (MBs) and fatty change [28–30]. For many years, MBs, hyaline inclusion body, were observed characteristically in alcoholic liver disease, but it was soon realized that MBs are not specific for this and may occur in other non-alcoholic and chronic liver diseases of animal models [29,30]. Recent reports have demonstrated that CK8 and CK18 were identified as major components of MBs in humans and experimental animals [22]. In the current investigation, a large number of CK 8/18-positive cells were detected around necrotic hepatocytes, especially in the TG-99 group.

In case of chronic liver disease, alcoholism and infection with HCV frequently coexist. It is widely believed that the combination of HCV infection and ethanol consumption may impair cellular immunity, and result in severe and progressive liver disease [14,15].

Piecemeal necrosis is characterized by the destruction of liver cells occurring in the presence of mononuclear inflammatory cell infiltration [31]. This process is repeated while the cytoplasm and nucleus of the hepatocyte disappear, until only residual cytoplasm of the hepatocytes remain either attached to intact hepatocytes or surrounded and sequestered within scar tissue and lymphocytes [31]. The role of liver cell surface antigen in other types of chronic liver disease where piecemeal necrosis is a prominent feature, such as alcoholic hepatitis and non-alcoholic steatohepatitis, is less understood [31]. In this study, piecemeal necrosis appeared to push into hepatocytes with lymphocytes and indent the liver cell cytoplasm. The outline of affected liver cells persisted even after the nucleus disappeared. Only cytoplasmic remnants could be found in piecemeal necrosis cells and dissociated from hepatocytes in necrotic central or pericentral vein areas.

HCV core protein expression has been associated with changes in the sensitivity of hepatocytes to apoptotic stimuli. Hepatocyte apoptosis promotes liver fibrosis, especially in patients with impaired liver regeneration [9]. HSCs are a source of collagen and other extracellular matrix proteins. These cells also generate collagenases, gelatinases, and tissue inhibitors of metalloproteinases [23]. Although activated HSCs are found in cases of both alcoholic and HCV-associated liver disease, different subpopulations are affected by alcohol and HCV. With alcohol consumption, fibrosis initially occurs in the central vein area whereas fibrosis develops in the periportal area in case of chronic HCV infection. HSCs are known to respond to and produce multiple cytokines [9].

TGF-β and platelet-derived growth factor (PDGF) are the two main cytokines produced by HSCs. TGF-β is the major stimulus for extracellular matrix overproduction whereas PDGF is a potent HSC mitogen [26,27]. TGF-β1 is produced by HSCs and has a dual impact on the progression of liver disease by promoting fibrogenesis and inducing hepatocytes apoptosis [5]. Smad is an intracellular signaling molecule belonging to the TGF-β superfamily that plays an important role in the activation of HSCs and hepatic fibrosis [23,24]. The TGF-β signaling cascade is initiated when TGF-β binds to the corresponding transmembrane receptors. The activated TGF-β receptors then phosphorylate and activate Smad proteins, which transduce the signal from the cytoplasm to the nucleus [32]. In the nucleus, Smad can bind directly to DNA and cooperate with other transcription factors to induce the transcription of target genes. Smad transactivation of p21 gene transcription occurs in the proximal promoter region [7]. The p21 protein is a universal CDK inhibitor and is regulated transcriptionally by p53 that is activated by DNA damage. Hepatocytes in chronic hepatitis get several DNA damage by lymphocytes and Kupffer cells [33].

p21 is a key mediator of cell cycle checkpoints and apoptosis [34]. This factor interacts with and inhibits different targets essential for cell cycle progression. p21 expression is up-regulated in response to numerous anti-proliferative factors [35]. p21 is involved in growth inhibition caused by irradiation [36], TGF [37], oxidative stress [38,39], differentiation factors [40], anti-neoplastic agent [41], and ethanol [42]. Kobayashi *et al*. [43] reported that p21 mRNA expression in normal tissues is significantly higher in HCV-positive cases compared to HBV-positive cases. These data supports our findings that p21 protein expression was affected not only by fibrosis but also hepatitis virus type. It is also possible that p21 expression accompanying chronic liver disease is closely related to subsequent carcinogenesis in liver because reduced p21 expression is associated with many types of cancers including HCC [44–46].

Also, HCV core gene plays dual function on p21. HCV core gene has two isoform. One is innate form which is located in cytoplasm and another is mature form which is in nucleus. Each isoform has distinct function on p21. The innate form in cytoplasm increase p21 and leads to arrest of cell cycle. On the other hand, mature form in nucleus decrease p21 and leads to activation of cell cycle. We assumed that in case of TG-99, the core gene cannot enter nucleus by inhibition the phosphorylation of Ser99. Moreover, the innate form which cannot enter nucleus and remain in cytoplasm would be increased. The innate form increases the expression of p21 [47,48].

In the other animal that we used, however, the core gene might be phosphorylated and the phosphorylated core gene can enter nucleus. It would then remain in its mature form, which does not increase or down-regulate p21. Also, TG-116 did not show overexpression as TG-99 although TG-116 has a mutated phosphorylation site like TG-99. We suggest that the phosphorylation site, serine 116, cannot affect subcellular localization like serine 99.

We performed Azan stain and immunohistochemistry of α-SMA to determine the extent of fibrosis in the different groups of mice. No significant fibrosis or MFBs were found in any of the animals. As an importan cytokine that promotes fibrosis, cells expressing TGF-β1 were clearly detected in the TG-99 mice but not in NTG, TG-K or TG-116 animals. Because alcohol intake and HCV induce ROS generation, possibly by promoting hypoxia, TGF-β1 was produced in hepatocyte of TG-99 mice. The expression of p-Smad2/3, a down-stream signaling factor of TGF-β1 [24,49], was detected more frequently in TG-99 animals compared to other groups of mice.

In general, increased TGF-beta activates HSC. The activated HSCs transdifferentiate to myofibroblasts which are the main source of collagen production and fibrosis in the end. However it is difficult to induce fibrosis in rodents since the reversion to normal liver is easy in rodents especially in mice. Researchers whose research target is hepatic fibrosis usually use CCl4 injection, which shows strong hepatotoxicity to induce fibrosis in rodents [50]. Although alcohol abuse is directly connected to hepatic fibrosis in the human case, in rodents, however, it is hard to get hepatic fibrosis with only *ad libitum* alcohol administration since their blood alcohol concentration is not sufficient to induce hepatic injuries and its rapid metabolic rate by liver phase I and II class enzymes. The metabolic rate of rodents is 4,5 fold higher than that of humans [51]. To induce hepatic fibrosis in rodents using alcohol, additional chemicals such as carbontetrachloride or penobarbital are necessary [52].

TG-99 showed more severe pathological liver changes including hepatic necrosis and hepatocyte dissociation when compared to non-transgenic mice, TG-K and TG-116. Also, TG-99 had much more positive cells for CYP2E1, CK8/18, TGF-β1, p-SMAD2/3 and p21. TG-99 is the mice model in which serine 99 is substituted with glutamine. Serine is known as the phosphorylation site of HCV core protein. Among many serine of HCV core protein, serine 99 is the main site that is phosphorylated by protein kinase C. Phosphorylated serine 99 plays a role in suppressive activity in HCV core protein [20]. TG-99 mice used in this study, however, could not have suppressive activity of HCV core protein since serine 99 is replaced with glutamine which leads to inhibition of phosphorylating serine 99. This would be the reason why TG-99 mice had more severe hepatic changes and vulnerable to alcohol induction.

However, both Serine 116 and Serine 99 is phosphorylation site of HCV core protein. The serine 99 is phosphorylated by PKC, while serine 116 is phosphorylated by PKA. The TG-99 is the mice model whose serine 99 is substituted by glutamine and the TG-116 is the mice model whose serine 116 is replaced by isoleucine. Thus protein kinase such as PKA and PKC cannot phosphorylate serine 116 or 99 in TG-116 and TG-99. Why does TG-99 show more severe hepatic changes when comparing to other groups? Although the phosphorylation is prevented in TG-116 like TG-99, TG-99 did not show severe hepatic injury in our study. Also, TG-116 did not show overexpression of p21. This means that inhibition of phosphorylation of serine 116 did not affect the subcellular localization. Moreover, TG-116 have more mature HCV core gene leading to stable or down-regulated p21 expression. Thus, TG-116 activates cell cycle of hepatocytes leading to active proliferation and recovery from hepatic injury.

Experimental evidence suggests that HCV and alcohol consumption exert additive inhibitory effects on antiviral immune response. In addition, specific pathways have been identified by which the HCV core protein and alcohol interact to activate hepatocytes. Nonspecific inflammatory cell recruitment and pro-inflammatory cytokine activation have also been implicated in the development of both alcohol- and HCV-induced liver diseases.

In summary, alcohol intake induced piecemeal necrosis and hepatocyte dissociation in HCV core mutant 99-Tg mice. These phenomena involved activation of the TGF-β1/p-Smad2/3/p21 signaling pathway in hepatocytes. Our investigation produced the data that will be useful for studying hepatic pathogenesis associated with alcohol and HCV in humans. Taken together, all these data indicate that the TG-99 mice showed significant hepatic patterns with alcohol consumption, suggesting these animals may represent a valuable model of hepatic degeneration that will help elucidate the mechanism underlying liver degeneration in patients infected with HCV who consume alcohol.

## 4. Experimental Section

### 4.1. Animals and Experimental Design

The transgenic mice used in this study were generated as previously described [19]. They were produced by the injection of the HCV core gene and its mutant genes under a transcriptional regulatory element from HBV enhancer into mouse embryos obtained from F1 hybrid of C57BL/6 and CBA. The TG-K expresses the wild type core gene in liver. The TG-99 transgenic mouse expresses a mutant core gene, in which serine-99 was substituted with glutamine (mutant serine(S) glutamine(Q)). The transgenic mouse line (TG-116) expresses mutant core gene, which serine-116 was substituted with isoleucine (mutant serine(S)→isoleucine(I)). Transgenic and wild type mice 4–5 months and weighing about 25 to 35 g were used for all experiments. The mice were divided into four groups: Wild-type mice (NTG; *n =* 11), TG-K mice (*n* = 11), TG-116 mice (*n* = 11), and TG-99 mice (*n* = 11). The mice had free access to drinking water containing 10% ethanol (Merck KGaA, 64271; Darmstadt, Germany) with 5% sucrose for 13 weeks. All mice were maintained in a room at 22 ± 2 °C with a relative humidity of 50% ± 10% and a 12 h light-dark cycle. All mice (*n* = 44) were sacrificed simultaneously after 13 weeks of ethanol supply.

### 4.2. Histopathologic and Immunohistochemistry Analyses

Pieces of the liver were collected from each mouse, fixed in 10% neutral buffered formalin, processed routinely and embedded in paraffin wax. Tissue sections were cut into 4 μm-thick pieces and stained with hematoxylin and eosin (H&E) as well as Azan stain for collagen fibers.

Immunohistochemical stainings were performed by the labeled streptavidin-biotin method using a Histostatin^®^-plus bulk kit (Zymed Laboratories Inc., San Francisco, CA, USA). The following primary antibodies were used: a monoclonal anti-α-smooth muscle actin (α-SMA) antibody at a 1:800 dilution (clone 1A4, Sigma Chemical Co., St Louis, MO, USA), anti-CYP2E1 antibody at a 1:200 dilution (Oxford Biomedical Research, Rochester Hills, MI, USA), a mouse monoclonal anti-cytokeratin 8/18 (CK 8/18) antibody at a 1:100 dilution (Novocastra Laboratories Ltd., Newcastle, UK), anti-transforming growth factor-β1 (TGF-β1) antibody at a 1:100 dilution (Santa Cruz Biotechnology, Santa Cruz, CA, USA), anti-phosphorylated (p)-Smad2/3 antibody at a 1:100 dilution (Santa Cruz Biotechnology) and anti-p21^WAF1^ (p21) antibody at a 1:100 dilution (Santa Cruz Biotechnology). After blocking endogenous peroxidase activity with 3% hydrogen peroxide in methanol at room temperature (RT), the sections were incubated with 10% non-immunized goat serum was applied to the sections to inhibit nonspecific reactions. The sections were then incubated with primary antibodies at 37 °C. Next, the sections were incubated with biotinylated secondary antibody at RT, exposed to peroxidase-labeled streptavidin at RT and finally incubated with 3,3-diaminobenzidine tetrahydrochloride (DAB) as a substrate. The sections were washed with PBS (pH 7.2) after each step. Counterstaining was carried out with Mayer’s hematoxylin. Non-immunized goat serum was substituted for the primary antibody as a negative control.

To quantify the immunohistochemistry staining, we randomly selected five microscopic fields (200×) of liver sections and counted the positive cells for each antibody.

### 4.3. Statistical Analysis

Statistical analysis was performed using one way analysis of variance (one way ANOVA) to compare multiple groups. All results are presented as the means ± S.E.M. The value of statistical significance was set at 0.01.

## 5. Conclusions

In this study, we reveal the synergistic effects of alcohol administration and HCV core protein. Also, we showed that the phosphorylation of Ser 99 in HCV core gene is important in progression of hepatic degeneration under hepatic injury such as alcohol administration.
